# Skin Tissue Substitutes and Biomaterial Risk Assessment and Testing

**DOI:** 10.3389/fbioe.2018.00086

**Published:** 2018-07-26

**Authors:** Houman Savoji, Brent Godau, Mohsen Sheikh Hassani, Mohsen Akbari

**Affiliations:** ^1^Institute of Biomaterials and Biomedical Engineering, University of Toronto, Toronto, ON, Canada; ^2^Toronto General Research Institute, University Health Network, University of Toronto, Toronto, ON, Canada; ^3^Laboratory for Innovations in Microengineering (LiME), Department of Mechanical Engineering, University of Victoria, Victoria, BC, Canada; ^4^Center for Biomedical Research, University of Victoria, Victoria, BC, Canada; ^5^Centre for Advanced Materials and Related Technology, University of Victoria, Victoria, BC, Canada; ^6^Department of Systems and Computer Engineering, Carleton University, Ottawa, ON, Canada

**Keywords:** wound healing, skin substitutes, biomaterials, immunomodulation, regulatory pathway

## Abstract

Tremendous progress has been made over the past few decades to develop skin substitutes for the management of acute and chronic wounds. With the advent of tissue engineering and the ability to combine advanced manufacturing technologies with biomaterials and cell culture systems, more biomimetic tissue constructs have been emerged. Synthetic and natural biomaterials are the main constituents of these skin-like constructs, which play a significant role in tissue grafting, the body's immune response, and the healing process. The act of implanting biomaterials into the human body is subject to the body's immune response, and the complex nature of the immune system involves many different cell types and biological processes that will ultimately determine the success of a skin graft. As such, a large body of recent studies has been focused on the evaluation of the performance and risk assessment of these substitutes. This review summarizes the past and present advances in *in vitro, in vivo* and clinical applications of tissue-engineered skins. We discuss the role of immunomodulatory biomaterials and biomaterials risk assessment in skin tissue engineering. We will finally offer a roadmap for regulating tissue engineered skin substitutes.

## Introduction

Skin is the largest organ in the human body and any damage to this living organ has dramatic and significant consequences which may lead to mortality, hospitalization or long-term morbidity (Korrapati et al., 2016). Tissue engineering is a promising and interdisciplinary active area of research in biomedical engineering that provides and investigates the application of novel biomaterials for the reconstruction of diseased or damaged tissues and organs (Chua et al., 2016). Tissue engineered skin substitutes provide new therapy potentials for treatment of acute and chronic skin wounds. Several important characteristic factors such as tunable physical, morphological and mechanical properties, suitable permeability, biocompatibility, non-toxicity and non-inflammatory; among others, need to be carefully considered in the fabrication of a functional skin substitute (Albanna and Holmes IV, 2016). In addition, a skin substitute should be able to replicate the gradients of various growth factors, cytokines, enzymes and pharmacological agents *in vivo* to promote optimal restoration and regeneration of full thickness wounds (Chua et al., 2016). For this purpose, scientists have used natural and synthetic polymers to mimic the natural extracellular matrix (ECM) and recapitulate the structure and function of the envisaged tissues (Korrapati et al., 2016).

Although recent advances in skin tissue engineering have offered potential to significantly improve the clinical outcome in wound healing of both acute and chronic wounds, there are still some deficiencies that need to be addressed to provide substitutes with painless healing process and encourage the formation of vascular, neural and lymphatic networks, hair follicles, sebaceous and sweat glands (Pereira et al., 2013). Therefore, the ultimate goal of these efforts in skin tissue engineering is to fabricate a complex scar-free skin substitute that can be transplanted in large quantities in only one surgical intervention with a minimum chance of rejection by the host's body.

This review will summarize the advances in the engineering of skin substitutes both *in vitro* and *in vivo*. We further discuss the role of immunomodulatory biomaterials and biomaterials risk assessment in skin tissue engineering. We will then offer a roadmap for biomaterial selection, risk assessment and testing of skin substitutes. Finally, we will discuss prospects for further progress in skin regeneration in the future.

## Skin anatomy and physiology

**Figure 1:**
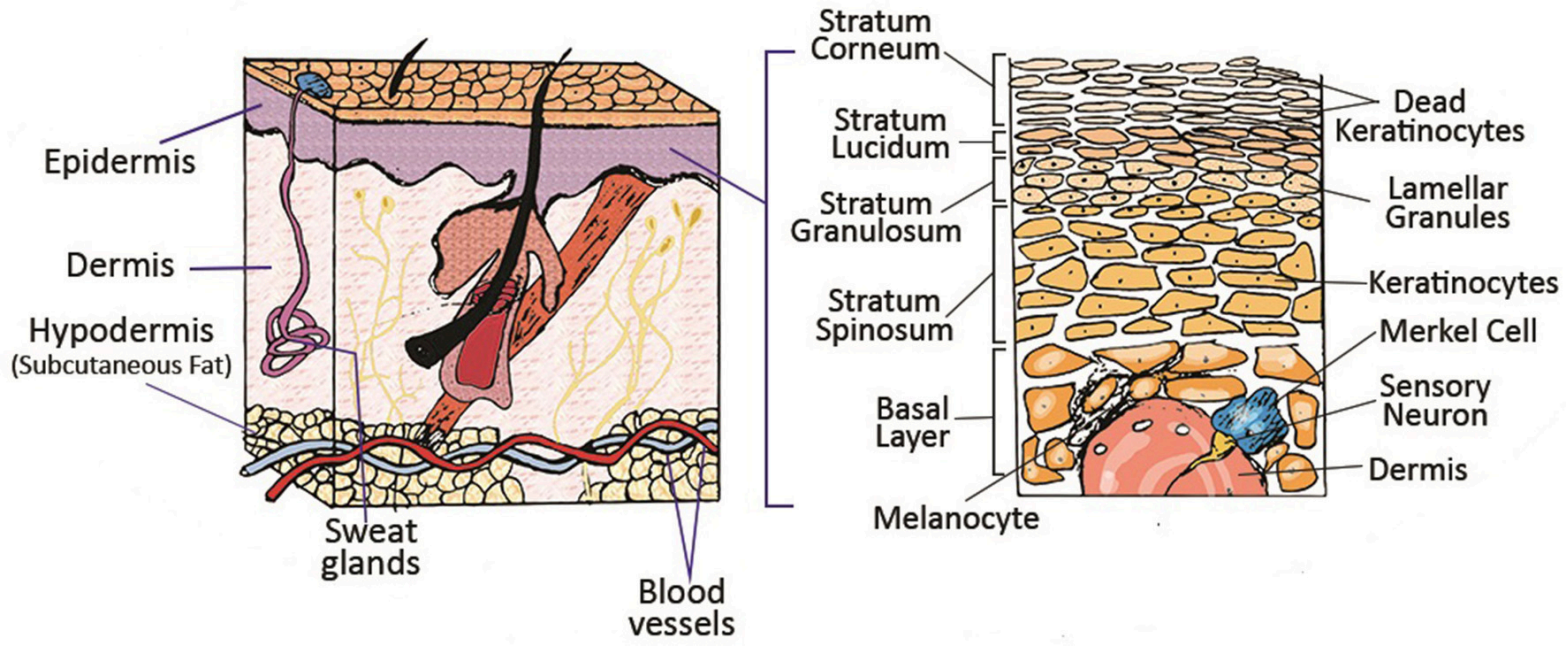
Schematic of different layers of skin and its appendages. Reprinted with permission from Mohammadi et al. (2016). Copyright 2018, John Wiley & Sons.

The skin is the largest organ of the human body, serving as an interface between the body and the surrounding environment. The primary function of this complex organ is to protect the internal organs against external insults such as pathogens, as well as thermal, mechanical and chemical hazards (Groeber et al., 2011). The skin is composed of different cells and multiple anatomically distinct layers, commonly classified into three main compartments; epidermis, dermis and subcutaneous tissue (hypodermis) (Figure 1). The epidermis is a dynamic, continuously self-renewing multilayered epithelium, mainly composed of keratinocytes. These keratinocytes have the ability to differentiate and undergo structural and compositional changes, leading to the synthesis and expression of a variety of structural proteins and lipids, therefore playing a vital role in skin function (Bouwstra et al., 2003). The epidermis can be subdivided into stratum corneum, stratum lucidum (only in some parts), stratum granulosum, stratum spinosum and stratum germinativum. The uppermost layer of the epidermis, the stratum corneum (SC), is a 10–20 μm thick layer of enucleated dead cells (corneocytes) embedded in a lipid matrix (Groen et al., 2011; Flaten et al., 2015). The lipid matrix, which mainly consists of ceramides, cholesterol and free fatty acids, is considered to play a central role in the barrier functionality of skin against absorption of components and water loss (Hatta et al., 2006; Masukawa et al., 2008). The epidermis is connected to its adjacent layer, the dermis, via the basal membrane. Hair follicles, sweat glands, shafts and nerves are all embedded in this sub-layer (Bouwstra and Ponec, 2006). The dermis is around 1–2 mm thick and provides mechanical support for the skin as well as the elastic properties due to the high amount of elastin in this layer. The dermis itself is comprised of a loosely arranged collagen fiber upper papillary layer and a dense collagen fiber reticular layer (Mathes et al., 2014). The hypodermis is the final sub-layer, which functions as the skin's shock-absorber and the body's heat insulator, and is mainly comprised of fibroblasts and adipocytes (Mathes et al., 2014).

## Etiology, pathology and pathophysiology of wounds

The skin covers about 3,000 square inches of the body surface and weighs around one-sixth of the entire body, thus it is the most exposed organ in the body to external hazards (Flora, 2002). Skin injuries are breaks in the skin tissue caused by surgical procedures, genetic irregularities and physical and chemical traumas. These wounds can also be divided into the following categories based on the depth of damage; epidermal, superficial partial-thickness, deep partial-thickness and full-thickness skin wounds (Papini, 2004). Epidermal and partial-thickness level wounds are normally regenerated using the skin's self-healing functions. However, in deep partial-thickness and full-thickness skin wounds, self-healing is not possible since the skin's epithelial regenerative elements are completely destroyed (Blanpain et al., 2004; Tumbar, 2006).

**Figure 2:**
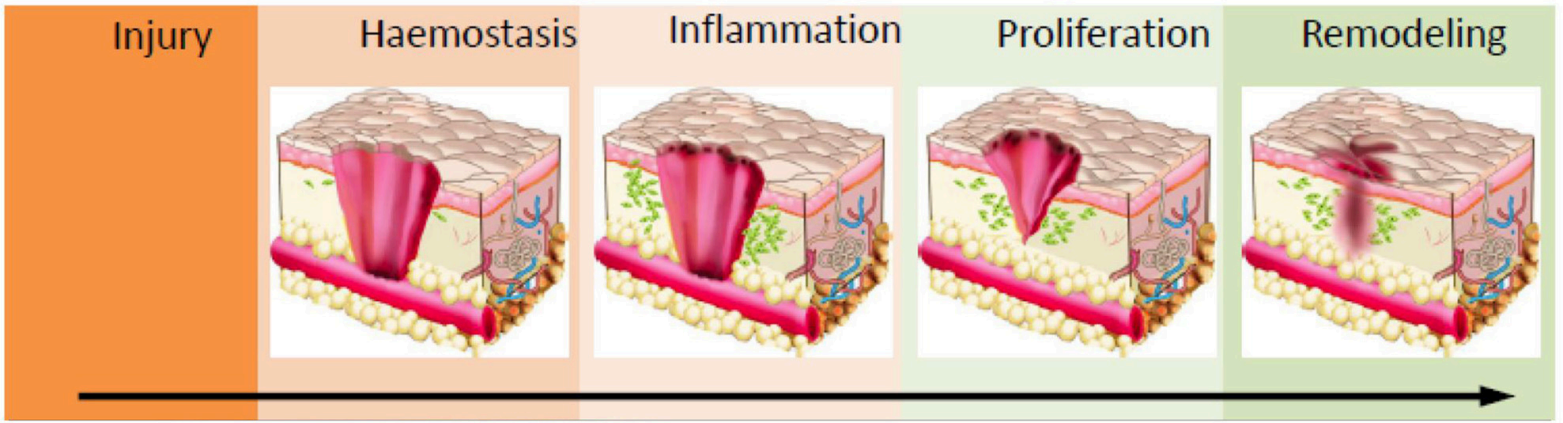
Schematic of the wound healing process. Haemostasis via coagulation and clot formation occurs at the wounded site followed by immune cells infiltration and inflammation to clean up the site of injured tissue. This prevents infection and triggers granulation. In the proliferation phase, fibroblast, epithelial cells, keratinocytes and endothelial cells will migrate and proliferate into wounds to deposit ECM proteins and other biomolecules which enables wound closure. Ultimately, in the maturation/remodelling phase, ECM deposition and clearance controls the development of scar formation. Reprinted with permission from Lin et al. (2018). Copyright 2018, MDPI.

Wound healing occurs in four concurring phases; hemostasis, inflammation, cell proliferation and remodeling (Figure 2) (Hu et al., 2014). Upon the infliction of the injury, the skin rapidly responds with a series of actions. Platelets stimulate the inflammatory response by releasing proteins and growth factors. The site of the injury immediately recruits immune cells into the wound, where the accumulation of the platelets results in blood coagulation to prevent blood loss (Midwood et al., 2004). Fibroblasts enter the wound site and generate new tissue matrix from fibronectin and collagen (Groeber et al., 2011). Subsequently, keratinocyte re-epithelialization and the revascularization of the damaged area occurs via endothelial cells, while concurrently fibroblasts differentiate into myofibroblasts to close the wound by shrinking the matrix (Midwood et al., 2004; Groeber et al., 2011). Finally, cells undergo apoptosis which results in scar tissue formation. This process gives the skin its remarkable regeneration capacity and enables it to maintain homeostasis in response to a variety of disturbances throughout our lifetime. This self-repair capability is in large due to the presence of epidermal stem cells in different compartments of the skin such as inter-follicular compartments and epidermal appendages (Mathes et al., 2014).

## Tissue-engineered human skin equivalents

The first milestone in skin tissue engineering was the in-lab culture of keratinocytes in 1966 (MacNeil, 2007). This led to the development of cultured epithelial autografts (CEAs), which consisted of small sheets containing two or three layers of cells (Vignesh, 1981; Gallico et al., 1984). The next important step was the design and *in vivo* evaluation of a dermo-epidermal skin substitute in human namely Apligraf^®;^, which was made of human allogeneic fibroblasts and keratinocytes (Bell et al., 1981). Later on, attempts were made to develop a skin substitute similar to Apligraf^®;^ by using human autologous keratinocytes and fibroblasts in bovine collagen and were applied to extensive burns and ulcers (MacNeil, 2007).

In 1981, another practical and major step in the tissue engineering of the skin was reported by designing a dermal substitute named Integra™, which comprised bovine collagen and shark chondroitin sulfate with a silicone membrane, acting as a temporary barrier. Practically, Integra™ was grafted to the wound site leading to formation of blood vessels. Then, the silicone barrier was removed and replaced with a layer of autologous cells (Burke et al., 1981). Several commercialized models have been marketed for permanent and temporary use in clinics during the last several decades. They are usually comprised from two compartments; biodegradable material as scaffolds (natural or synthetic polymers) which are used to support cell attachment, and cells which could be autologous, allogenic or xenogeneic. These commercially available skin substitutes are categorized into three main products namely, epidermal, dermal and dermo-epidermal substitutes. In this section, we briefly discuss some of these substitutes and their pros and cons in wound repair and regeneration.

## Epidermal substitutes

Inspired by CEAs, these substitutes have a small stratified sheet of cells (i.e., autologous keratinocytes which are grown in the presence of murine fibroblast). *In vitro* culture of autologous cells is performed by skin biopsy (approximately 2–5 cm^2^). Single keratinocytes are extracted and cultured to form colonies (Gallico et al., 1984). The single colonies come together to form stratified epithelial layers and eventually these layers are delivered to the wound site. This process takes 3–4 weeks upon the patient's arrival at the clinic. Epicel®, Epidex™ and Myskin™ are some of the examples of these substitutes (Wood et al., 2006). Despite the shortcomings of these products, they have still been applied for patients with extensive burns/wounds (Atiyeh and Costagliola, 2007). Epicel® is prepared using autologous keratinocytes which form the CEA sheets 15 days after skin biopsy (Vacher, 2003), whereas Epidex™ is cultured from keratinocytes obtained from the outer root sheath of scalp hair follicles (Tausche et al., 2003). Myskin™ is made up of a surface coated silicon substrate, covered with sub-confluent autologous keratinocytes which improves handling application and decreases the cell culture time. This product was reported to treat diabetic foot ulcers and superficial burns (Moustafa et al., 2007). The main disadvantages of epidermal substitutes are their long preparation time, poor keratinocyte attachment, difficult handling due to the thin cellular layers, poor mechanical stability, scarring and their high production costs (Atiyeh and Costagliola, 2007).

In another approach [i.e., ReCell®, (CellSpray)], the suspended cultured autologous keratinocytes are directly sprayed onto the wound site. This method showed faster formation of the epidermis layer in *in vivo* wound models but human application remains controversial (Navarro et al., 2000). The advantages of this approach include accelerated healing, minimizing scar formation, eliminating tissue rejection and re-introducing pigmentation to the skin. However, use of different scaffolds (fibrin matrix, silicon, etc.) has definite effects on shortening the fabrication process of epidermal substitutes and increasing the surface area of CEAs (Ronfard et al., 2000).

## Dermal substitutes

Engineered dermal substitutes provide appropriate configuration and surface area for an effective epidermal engraftment. Several *in vitro* and clinical trials have shown successful engraftment of cultured autologous keratinocytes when applied to the dermal or neo-dermal bed (Hansbrough et al., 1993; Wood et al., 2006; Pham et al., 2007). Most of the dermal substitutes contain a matrix without incorporating cells and are applied permanently to the wound bed (Wood et al., 2006; Pham et al., 2007). Some currently commercially available dermal substitutes are AlloDerm®, Dermagraft®, Integra™, and Matriderm®; among others. AlloDerm® is an acellular human dermis which is produced by the removal of the epidermis and extraction of fibroblasts from the dermis while the collagen bundles or the basement membrane remains unchanged (Shakespeare, 2005). This product does not cause immunogenic response due to its acellular structure.

Dermagraft® is an engineered dermal substitute which contains cryopreserved human fibroblast cells derived from newborn foreskin tissue. The human neonatal fibroblasts are seeded onto a biodegradable polyglactin mesh scaffold. The fibroblasts proliferate to fill the pores of this scaffold and release human dermal collagen, matrix proteins, growth factors and cytokines to form a 3D human dermal substitute containing metabolically active living cells. Dermagraft® does not include macrophages, lymphocytes, blood vessels or hair follicles. It can promote re-epithelialization in the restoration of the dermal bed and wound healing especially in diabetic and venous ulcers (Gentzkow et al., 1996). Cost and antigenic response are the main disadvantages of this graft (Gentzkow et al., 1996).

Integra™ is the first approved tissue engineered product by the U.S. Food and Drug Administration (FDA) to regenerate dermis. This substitute consists of a porous matrix of cross-linked bovine type I tendon collagen, shark chondroitin-6-sulfate glycosaminoglycan and a semi-permeable polysiloxane. The semi-permeable silicone membrane controls water vapor loss, provides a flexible anti-bacterial support for the wound surface and promotes enhanced mechanical strength for the substitute. On the other hand, the collagen-glycosaminoglycan biodegradable matrix provides a scaffold for cellular invasion/infiltration and capillary growth (i.e., vascularization). Once applied, the infiltration of fibroblasts into the scaffold is inhibited, resulting in neo-dermis formation. After the completion of vascularization and neo-dermis formation (approximately 15–20 days), the silicone layer is peeled off and the wound can be closed permanently with an epidermal substitute. Integra™ provides patients with several promising advantages including long shelf life, simple handling, comfortability for various anatomical sites, excellent performance in deep donor sites, low risks of immunogenic response and disease transmission and reduced rates of contraction and scarring. It could be applied for a wide range of treatments including full-thickness burns, chronic ulcer and full-thickness non-thermal skin wound management; among others (Bello et al., 2001).

Matriderm® was designed as a 3D matrix consisting of collagen matrix coated with an elastin hydrolysate from the ligament, similar to the structure of the human dermis. The collagen matrix acts as a supportive structure for the growth of living cells and blood vessels. The elastin component promotes the stability and elasticity of the regenerating tissue. During the healing process, fibroblasts produce their own ECM, and the scaffold is resorbed. Matriderm® possesses more elastic properties similar to that of natural skin and can be applied in a single stage process which eventually reduces scar formation and wound contraction (Ryssel et al., 2008).

## Dermo-epidermal substitutes

Dermo-epidemal substitutes (composite skin substitutes) are comprised of two layers including keratinocytes on fibroblast-containing dermal substitutes. The cells could be autologous and allogeneic skin cells (i.e., keratinocytes and fibroblasts), which are integrated into scaffolds. However, using allogeneic skin cells is controversial due to the host body rejection. They are the most advanced skin substitutes which faithfully mimic both epidermal and dermal layers. Providing growth factors, cytokines and ECM for host cells, initiating/regulating wound healing and effective pain relief are the advantages of these products. Although they can mimic the normal skin, they suffer from various shortcomings such as high costs, short shelf life and chance of tissue rejection by the host body (Shevchenko et al., 2010).

Apligraf^®;^ consists of two layers; the lower dermal layer contains bovine type I collagen and allogeneic neonatal fibroblasts, which produce additional matrix proteins. The upper epidermal layer is made of allogeneic neonatal keratinocytes. These layers form a substitute similar to normal human skin. It promotes transferring ECM components, cytokines and growth factors to the wound bed. Due to the short survival of the allogeneic cells (1–2 months), it can be applied as a temporary wound dress rather than permanent skin substitute (Griffiths et al., 2004). It is the FDA approved composite substitute to heal both diabetic foot ulcers and venous leg ulcers. Some efforts have been made to solve the shortcomings of using allogeneic cells by means of autologous cells but further clinical studies need to be done to confirm these results (Hernon et al., 2007).

OrCel® is a bilayered cellular matrix similar to Apligraf^®;^ in which normal human neonatal foreskin allogeneic epidermal keratinocytes and dermal fibroblasts are cultured in two separate layers into a Type I bovine collagen sponge. Donor dermal fibroblasts are cultured on and within the porous sponge side of the collagen matrix while keratinocytes, from the same donor, are cultured on the coated, non-porous side of the collagen matrix. OrCel® is comprised of an absorbable biocompatible matrix which has been shown to contain the cytokines and growth factors that are all suitable for host cell migration and wound healing. The extracellular secretion of cytokines and growth factors by the seeded cells is the main key factor to promote wound healing. It is applied for permanent skin replacement in severe burn patients. The clinical trials for this substitute demonstrated less scar formation and a shorter healing time when compared with the acellular bioactive wound dressing (Biobrane-L) (MacNeil, 2007).

## Emerging fabrication strategies for skin tissue engineering

Scaffolds are the backbones of any tissue-engineered skin substitute. They provide a platform for cells during the healing process. The structure, morphology, surface topography and mechanical elasticity of scaffolds play a crucial role in cell metabolic activities (e.g., cell-adhesion, -proliferation, -growth, and -differentiation) for successful neovascularization and complete wound repair. Traditional methods such as solvent casting/particulate leaching, freeze-drying (lyophilization), gas foaming, electrospinning, micro-patterning and micro-molding have been widely used for the fabrication of bioengineered tissue substitutes (Ma et al., 2003; Savoji et al., 2014a, 2016; Thadavirul et al., 2014; Limongi et al., 2015; Monteiro et al., 2015; Poursamar et al., 2015; Hadjizadeh et al., 2016; Ng et al., 2016; Mahmoudi et al., 2017). Recently, advanced biofabrication strategies such as three-dimensional (3D) bioprinting and biotextile have emerged as powerful tools that enable exquisite control over the micro and cytostructure of the bioengineering skin tissues (Akbari et al., 2016; Mirani et al., 2017; Pedde et al., 2017). In this section, we will focus on electrospinning, 3D bioprinting and biotextile as the three most popular biofabrication strategies for creating bioengineered skins substitutes.

**Figure 3:**
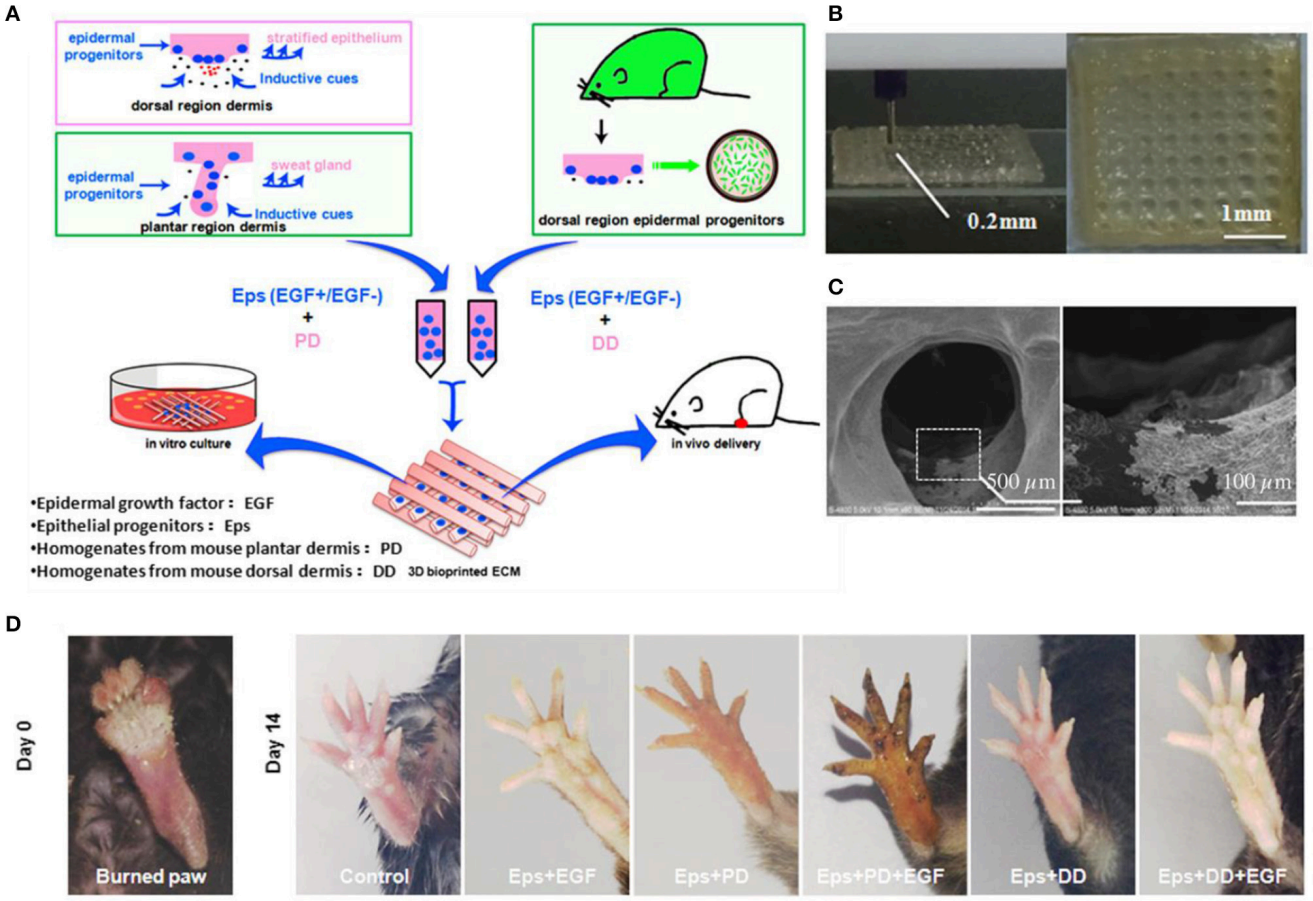
3D bioprinted scaffolds to direct the differentiation of epithelial progenitors for sweat gland regeneration. **(A)** Schematic of the printing process with epidermal progenitors and ECM incorporated in composite hydrogels. **(B)** Fabricated scaffold and **(C)** scanning electron microscopy (SEM) image of epidermal progenitor cells attached and spread out into the scaffold pores. **(D)** Iodine/starch-based sweat test on paws of mice at day 14 after surgery. Reprinted with permission from Huang et al. (2016). Copyright 2016, Elsevier.

**Figure 4:**
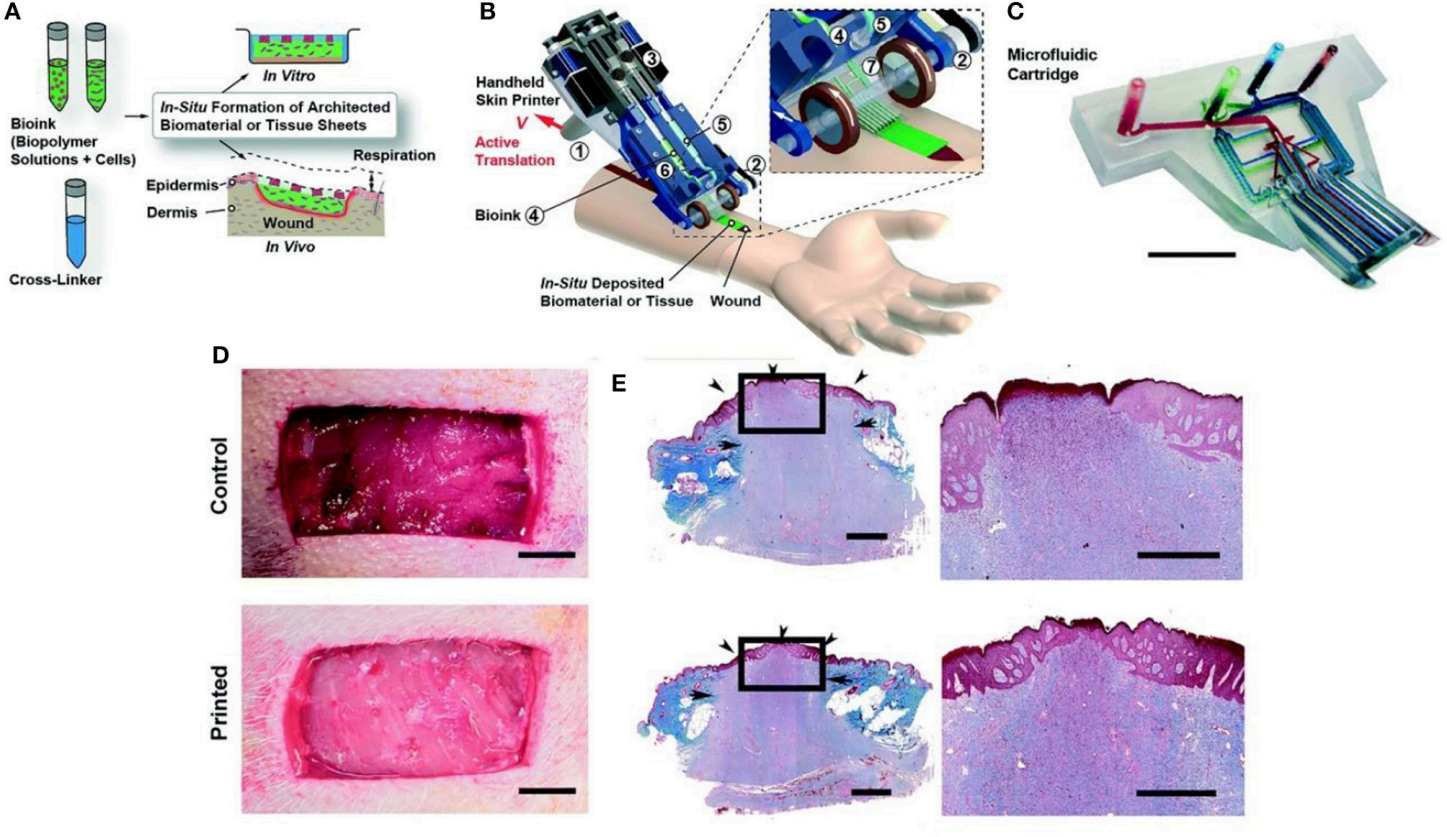
Handheld skin microfluidic printer. **(A)** Schematic diagram illustrating working principle of handheld bioprinter. Bioinks (green) containing hydrogels and cells, and a cross-linker solution (blue) are prepared. **(B)** Schematic image of handheld bioprinter. **(C)** Image of 3D printed microfluidic cartridge. Scale bar 10 mm. **(D)** Control wound and in situ printed of biomaterial sheet. **(E)** Granulation tissue formation and re-epithelialization confirmed by trichrome staining. Arrows indicate the border between newly formed granulation tissue and intact skin. Arrowheads mark epithelialized area. Arrowhead at the center of treated wound shows complete re-epithelialization, while central arrowhead in control wound shows non re-epithelialized zone at wound center. Scale bars 10 mm **(C, D)**, 1 mm (**E** right), and 2 mm (**E** left). Reprinted with permission from Hakimi et al., 2018. Copyright 2018, Royal Society of Chemistry.

3D bioprinting refers to the layer-by-layer deposition of biomaterials, bioactive molecules and living cells, on a 3D controllable platform (Pedde et al., 2017). The fabrication of 3D structures with complex geometries by 3D printing have been recently used in tissue engineering of the skin (Ng et al., 2016). The precise positioning with spatial control of bioactive substances enabled bioengineers to fabricate functional skin constructs with structural, biological and mechanical properties that are similar to those of the native skin (Pedde et al., 2017). The commonly used technologies for 3D printing and patterning of biological materials are inkjet, micro-extrusion, laser-assisted and microfluidic printing (Huang et al., 2017; Pedde et al., 2017; Hakimi et al., 2018). The selection of appropriate materials for use in 3D printing and their performance in a particular application depends on several factors including printability, biocompatibility, degradation kinetics and by-products, and structural and mechanical properties (Pedde et al., 2017). For example, 3D bioprinting was used to fabricate dermo-epidermal substitutes by printing a mixture of primary human dermal fibroblasts in a printable ECM-like bioink which were then seeded by primary human dermal keratinocytes (Rimann et al., 2016). The printed substitutes resulted in the formation of two-layer constructs containing distinct dermal and epidermal layers, suggesting the feasibility of 3D printed skin grafts. However, a fully stratified epidermis was not accomplished. (Rimann et al., 2016). 3D printing has also been utilized to fabricate full thickness skin constructs containing skin appendages (e.g., sweat gland; Huang et al., 2016). Mouse epidermal progenitor cells and suitable growth factors were encapsulated in gelatin and sodium alginate mixture as a bioink. The results revealed the successful differentiation of progenitor cells to sweat gland cells inside the ECM-like 3D printed structure. *In vivo* study in a small animal model (e.g., mice with severely burned paws) showed full regeneration of the functional sweat glands in animals (Figure 3). More recently, a handheld skin microfluidic-based printer was developed for *in situ* printing of biomaterial and skin tissue sheets containing dermal and epidermal cells embedded in different biomaterials (alginate, fibrin, collagen type I and hyaluronic acid) (Hakimi et al., 2018). *In vivo* results on a porcine full thickness wound model showed the feasibility of using this device for *in situ* biopolymer sheet deposition in a clinically relevant setting (Hakimi et al., 2018). H&E staining on healed wounds showed that both treated and control wounds formed complete granulation tissue, and displayed comparable levels of collagen deposition and cellularity (Figure 4; Hakimi et al., 2018).

Scaffolds/patches that mimic mechanical and morphological properties of native tissue, and that possess similar 3D fibrous structure and porosity, can be produced by a versatile electrospinning technique which has remarkably high controllability to tune the fibers architecture. Those fibrous structures undergo long periods of incubation because manual cell seeding is not uniform and cell infiltration is not complete over the entire depth of the scaffold (Savoji et al., 2014a, 2016; Hadjizadeh et al., 2016). Therefore, a novel approach has been investigated to spin cells-polymer solution in a single step, so called cell-electrospinning (Townsend-Nicholson and Jayasinghe, 2006). This fact could advantageously be used for regenerating 3D skin constructs by integrating autologous cells with these robust, tissue-engineered patches. Although there are several studies that have reported the high viability of the cells in a high electric field (Sampson et al., 2014), more investigation is needed to shed light on the precise assessment of cellular genetic change.

**Figure 5:**
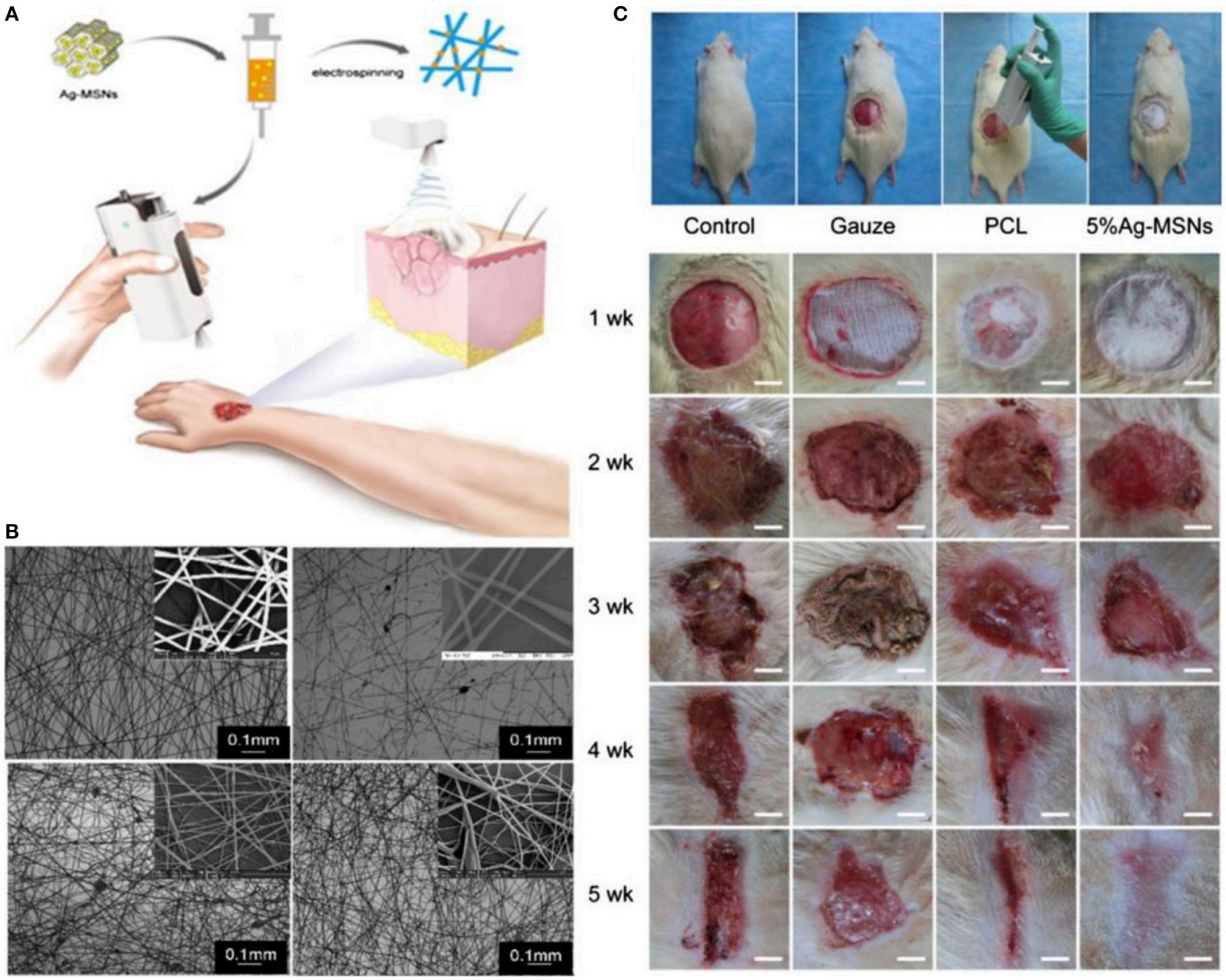
The use of *in situ* electrospinning process for wound management. **(A)** Schematic of the process showing the handheld electrospinning system. **(B)** SEM image of electrospun mats made by the handheld system. **(C)** *In vivo* evaluation of in situ electrospun mats, polycaprolactone (PCL), Ag-MSNs -mesoporous silica nanoparticles. Reprinted with permission from Xu et al. (2015) and (Dong et al., 2016). Copyright 2015 and 2016, Royal Society of Chemistry.

Another novel, easy and quick concept in wound healing is *in situ* electrospinning to fabricate suitable substitutes with or without encapsulated cells directly on the wounds. For example, a handheld portable electrospinning device for *in situ* electrospinning has been designed (Figure 5; Xu et al., 2015). The *in vitro* and *in vivo* results confirmed the antibacterial properties of the mesoporous silica nanoparticles dispersed in polycaprolactone (PCL) electrospun fibrous mats. Significant improvement of *in vivo* wound closure and re-epithelialization was observed 4 weeks after *in situ* treatment (Dong et al., 2016).

Biotextiles technologies including weaving, knitting, braiding and embroidering have also been investigated in skin tissue engineering applications to address the issues with permeability, mechanical strength and elasticity (Tamayol et al., 2013; Akbari et al., 2016). Various skin patches with significant permeability using natural and synthetic hydrogels have been reported with tunable structural, mechanical and biological properties (Grover and Tomar, 2016; Lu et al., 2017). For example, skin patches using collagen-laden poly(lactic-co-glycolic acid) and poly(lactic acid-co-caprolactone) incorporated with growth factors and bioactive molecules among others have shown promising outcomes in wound healing and regeneration (Townsend-Nicholson and Jayasinghe, 2006).

In addition to these emerging technologies which are combined with biochemical and biophysical cues in the skin substitutes' matrices (Xiao et al., 2017), commercially available therapies are still being used in clinics for treatment of diabetic wounds; for example, topical negative pressure (e.g., vacuum-assisted wound closure) (Lone et al., 2014), electroporation technique (Rouabhia et al., 2013; Snyder et al., 2017) and pulsed electromagnetic therapy (Choi et al., 2018).

## Immunomodulatory biomaterials for skin tissue engineering

The act of implanting biomaterials into the human body is subject to the body's immune response, and the complex nature of the immune system involves many different cell types and biological processes that will ultimately determine the success of a skin implant. Following implantation, the immune response can be categorized into three major phases in which the innate response acts on the order of days, the adaptive response acts on the order of weeks, and resolution occurs on the order of months (Chung et al., 2017). The underlying strategy in immunomodulation for regenerative medicine is to harness pro-regenerative cell types and biological functions that will not result in an inflammatory response and avoid foreign body giant cell formation. For current commercially available skin substitutes (e.g., Allografts, Dermagraft^®;^, Apligraft^®;^, and Transcyte^®;^), immunosuppressive drugs are often paired with implantation to avoid rejection of the implant (Skardal et al., 2012). The use of immunomodulatory biomaterials in an implant localizes immunosuppression to the wound site by removing the need for immunosuppressive drugs while having the potential to further reduce poor cosmetic outcomes. Common strategies in immunomodulation for skin regeneration include macrophage polarization (Sun, 2017; Castellano et al., 2018), the use of glycosaminoglycans (GAGs) (Bhowmick et al., 2017; Pezeshki-Modaress et al., 2017), and the use of decellularized matrices (Kuna et al., 2017).

**Figure 6:**
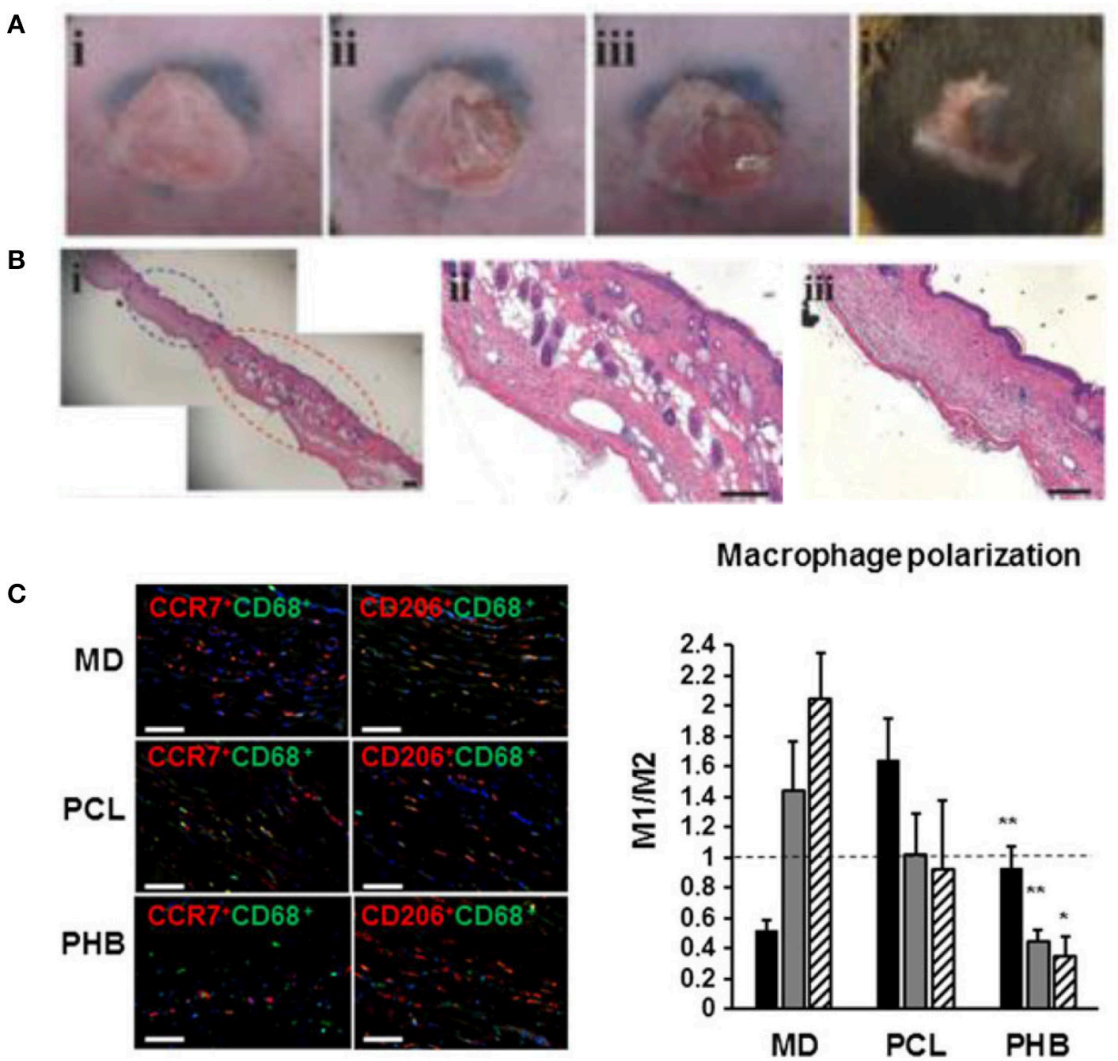
Immunomodulating biomaterials are effective in wound healing applications. **(A)** Creation of scar and treatment with immunomodulating DexIEME hydrogel. (i) Scar created by third degree burn, (ii) partial excision of scarred skin, (iii) apply hydrogel, (iv) wound healed after 5 weeks. **(B)** (i) H&E Stained scarred and regenerated skin, (ii) regenerated skin shows development of hair follicles, (iii) scarred skin lacking normal skin structure. Scale bars = 200 μm. Reprinted with permission from Sun (2017). Copyright 2017, John Wiley & Sons. **(C)** Macrophage polarization employed by treatment with Matriderm® (MD), PCL scaffolds, and PHB electrospun scaffolds. Nuclei were stained with DAPI (blue). M1(CCR7^+^CD68^+^)/M2(CD206^+^CD68^+^) ratio of macrophages present in the scaffold surrounding region 14 days following implantation. Scale bars = 50 μm (^*^p < 0.05, ^**^p < 0.01). Reprinted with permission from Castellano et al. (2018). Copyright 2018, John Wiley & Sons.

Macrophages, mature myeloid cells differentiated from circulating monocytes, display a range of phenotypes varying from the M1, pro-inflammatory type to the M2, pro-regenerative type (Rodero and Khosrotehrani, 2010). Their sensitivity to stimuli and ubiquity in immune processes makes them a prime target for strategic immunomodulation, with polarization to the M2 type being the goal. For example, dextran-isocyanatoethyl methacrylate-ethylamine (DexIEME) used as a hydrogel scaffold for cutaneous wound healing was shown to be effective in treating both pre-existing scars in mice and deep wounds in porcine animal models by promoting M2 macrophage polarization (Sun, 2017). DexIEME first led to differentiation of monocytes into macrophages followed by further polarization of differentiated macrophages to the M2 phenotype *in vitro*. This resulted in full skin regeneration *in vivo* after 5 weeks with ~75% of skin containing hair follicles when treating mice with third degree burn scars (Figures 6A,B). When treating deep wounds in porcine models, the hydrogel treatment showed full regeneration of skin with a reduction in fibrosis and the regenerated skin retains a reticulated endothelial layer. In another study, Castellano et al. showed a significant reduction in the M1/M2 ratio of biopsied tissue of mice implanted with electrospun poly (hydroxybutarate) (PHB) scaffolds when compared to MatriDerm® and PCL implants (Figure 6C; Castellano et al., 2018). The implant developed in this study was a dermo-epidermal skin equivalent in which human fibroblasts or endothelial cells were seeded and grown in the scaffold before xenograft implantation onto mice.

Glycosaminoglycans (GAGs) are long, linear polysaccharides that populate the ECM of the dermis and are important in promoting tissue regeneration in the wound healing process because they modulate the attraction of skin precursor cells (Ansari et al., 2018). Incorporation of GAGs into biomaterials has been shown to improve wound healing and promote a pro-regenerative environment in the wound. For example, chondroitin sulfate (CS), a major GAG, blended with gelatin and electrospun into scaffolds with varying ratios of gelatin to CS was shown to increase human dermal fibroblast (HDF) proliferation with increasing ratios of CS in the scaffold (Pezeshki-Modaress et al., 2017). The acellular and HDF seeded scaffolds were then implanted in excised rat skin wounds and showed reduced inflammation, complete re-epithelialization, and acceleration of wound healing with a reduction in fibrosis seen from the acellular scaffold to the HDF seeded scaffold.

Further strategies in immunomodulatory biomaterials include the use of decellularized ECM to reduce inflammation and promote a pro-regenerative host response. The major benefit of using decellularized ECM as a tissue scaffold is that the ECM inherently has a set of biomolecules that are naturally involved in the wound healing process. For example, decellularized pig skin was prepared as a gel with hyaluronic acid (HA) (Kuna et al., 2017). The gel, which contained 66.6, 3.5, and 4.6 μg/mL of collagen, elastin, and GAGs, respectively, showed a marked improvement in wound healing in nude mice by promoting rapid infiltration of host cells and improved wound stabilization. The gel was later mixed with human peripheral blood mononuclear cells before treatment to further improve the gels ability to promote neovascularization, resulting in improved wound healing capability. With an understanding of how the regenerative wound healing process works and how biomaterials can be modified to promote an anti-inflammatory and pro-regenerative environment in the wound, there is great potential to significantly reduce fibrosis and improve the cosmetic features of implanted skin grafts.

## Biomaterial risk assessment for skin tissue engineering

Due to the expensive cost of toxicological studies and difficulty of clinical trials to assess safety and efficacy, it is often easier to repurpose previously approved biomaterials for new applications. Nevertheless, there is a need for new, smarter biomaterials to improve the regenerative ability of future treatments in wound healing. The development of biomaterials for skin tissue engineering must be conducted with a desired end application and clinical trials in mind. In other words, rigorous standardized testing to consider a materials biocompatibility, toxicity, and long-term effects before going into clinical trials must be conducted. This requires not only a significant characterization of a biomaterials benefits for its' desired application, but also intelligent experimental design to disprove any potential safety concerns of the biomaterial.

**Figure 7:**
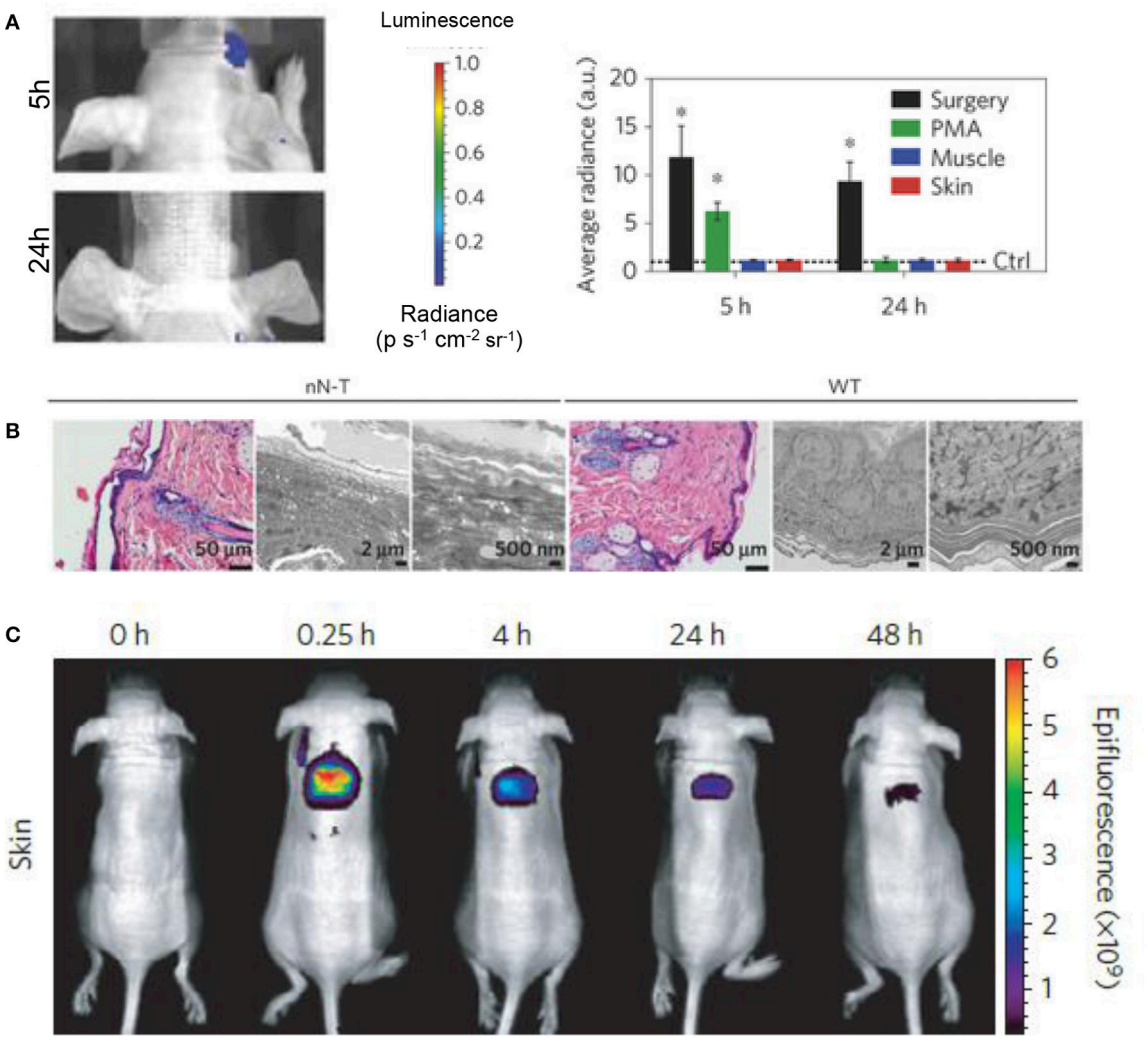
Biomarkers and fluorescent dyes are beneficial in biomaterial risk assessment. **(A)** Imaging and quantification of the luminol luminescence in mice treated with silicon nanoneedles. Phorbol-12-myristate-13-acetate (PMA) treatment and surgical incisions were employed as positive controls for acute inflammation and the data was normalized to the control represented by the dashed line (^*^p < 0.05, *n* = 3). Silicon nanoneedle treatment showed no acute inflammatory response in muscle or skin at 5 and 24 h. **(B)** H&E and transmission electron microscopy (TEM) micrographs of nanoneedle treated (nN-T) and wild-type (WT), or control, tissues show complex structure regenerated by the nanoneedle treatment. **(C)** Localized drug distribution in skin over 48h was shown by delivering fluorescent dyes with silicon nanoneedles. Reprinted with permission from Chiappini et al. (2015). Copyright 2015, Nature publishing Groups.

Chiapinni et al. developed biodegradable silicon nanoneedles that have the ability to induce neovascularization *in vivo* by delivering nucleic acids to skin (Chiappini et al., 2015). Before moving to *in vivo* studies, the cytotoxicity of their treatment was assessed *in vitro* with HeLa cells, showing that their treatment was not cytotoxic when compared to a control with an MTT proliferation assay. They paired this cytotoxicity assessment with an *in vitro* degradation test to exemplify that their biomaterial would not remain in the skin for long periods of time after treatment. They displayed the *in vivo* drug distribution by delivering fluorescent dyes with the nanoneedles and tracking the dyes over time to show local treatment with their system. In order to assess any potential acute inflammation from their treatment, real-time bioluminescent imaging was employed with administration of luminol, a compound which reacts with superoxides generated during acute inflammation to emit light (Figure 7; Gross et al., 2009). They further compared treated tissues with haematoxylin and eosin (H&E) histology to show intact tissue membranes after injection along with preserved epidermis, dermis, and sebaceous gland structure (Chiappini et al., 2015). They also observed no sign of leukocyte infiltration or capillary vessel disruption, and negligible hyperkeratosis and necrotic keratinocytes. A similar risk assessment was conducted for a urea based-bolaamphiphile injectable hydrogel with the potential use in skin tissue regeneration (Ramin et al., 2017). They noted the importance of their material to elicit a limited chronic inflammatory reaction, reduce fibrosis, and degrade on a optimal time scale for tissue regeneration. In order to exemplify limited chronic inflammation *in vivo*, they used lucigenin, a reagent which produces light when activated by reactive oxygen species produced by macrophages in chronic inflammation. Fibrosis was monitored via histological analysis with Masson's Trichrome staining, and *in vivo* degradation was analyzed by incorporating cyanin dye into their hydrogel and showing the decrease in fluorescence over a period of 21 days.

Presently, there is a move toward the use of more efficient and accurate evaluation systems for toxicity and inflammatory response. A more complete risk assessment can be conducted with the involvement of organ-on-a-chip systems and *in silico* studies. Organ-on-a-chip systems are a viable alternative to animal testing and can have strong predictive power on how human cells will react to biomaterials, which is a major limitation of animal models (O'Neill et al., 2008; Mohammadi et al., 2016). They also have the ability to be used for high-throughput screening of new biomaterials with a major cost-benefit. Modelling and simulations, or *in silico* studies, also provide valid arguments for new strategies in regenerative medicine, such as the argument made by Yannas et al. that the regeneration of injured skin is dependent upon the wound contraction process, rather than the scar formation process (Yannas et al., 2017). In this study, they suggest the use of a collagen scaffold in the wound healing process by determining the physical characteristics for optimized wound healing conditions and matching a biomaterial with those characteristics.

A combination of *in vitro, in vivo*, and *in silico* risk assessment will significantly strengthen the case for using new biomaterials in skin tissue regeneration. Extensive risk assessment in combination with strong consideration of the clinical hurdles to be encountered will facilitate the development of a new biomaterial, or an innovated old biomaterial, into stage I clinical trials.

### *In Vitro* models for skin substitute testing

Substantial efforts have been made in recent years to model and create substitutes that mimic human skin, placing the skin amongst the most developed *in vitro* engineered constructs. Since it is the body's first barrier being exposed to many types of cosmetics and therapeutics, extensive funding has been allocated by different industries for *in vitro* skin modulation in an effort to end continuous legal and ethical issues regarding product testing on skin (Karimi et al., 2016; Geraili et al., 2017). The *in vitro* modeling of the human skin can be divided into two main streams in terms of research motivation (Mathes et al., 2014). One stream places focus on obtaining deeper insight into the physiology of skin, requiring more complex models of the skin to further understand skin homeostasis. This branch places emphasis on studying transdermal drug administration and development of skin diseases for therapeutic intervention. Considering how common transdermal drug therapy is, it is necessary to optimize the drug delivery mechanism though the skin in order to enhance the outcome of the therapy (Savoji et al., 2014b; Flaten et al., 2015). This of course requires complex and robust predictive models which can realistically mimic the skin's intrinsic properties. The other branch focuses on developing validated *in vitro* skin models for risk assessment and toxicological screening. Strict legal and ethical restrictions on animal and human skin use and testing have created the basis for advancements in this branch, which in turn have led to the creation of optimal skin models (Hewitt et al., 2013). As a whole, these efforts have led to sophisticated *in vitro* skin models which are widely used for clinical applications, advancements in wound healing and as a test system for pharmaceutical and cosmetic research (Xie et al., 2010).

*In vitro* skin substitutes use 3D arranged human cells to mimic cell-cell and cell-matrix interactions. Most developed models are intended toward modeling the healthy skin with intact barrier properties, with only a few models mimicking the compromised skin. *In vitro* models can be broadly categorized into lipid and non-lipid based model membranes. Non-lipid based models are mainly silicon model membranes, which are used in a wide variety of studies to evaluate different methods and mechanisms of drug transport across the skin (Watkinson et al., 2009; Oliveira et al., 2011, 2012). A diverse range of lipid based models have also been developed. Kansy et al. developed a poly(2-acrylamido-2-methyl-1-propanamide) (PAMPA) (Kansy et al., 1998) membrane containing a phosphatidylcholine coated hydrophobic filter as a membrane barrier, and Sinko and his group enhanced the model to create the skin-PAMPA (Sink et al., 2012) containing synthetic certramides as a replacement for naturally existing ceramides in SC. Tsinman and Sinko further modified skin-PAMPA to predict skin penetration and screen topical formulations (silicone-based gel, silicone and acrylic copolymer) (Tsinman and Sinko, 2013). PVPA is another model designed to mimic the cells of biological barriers using a tight liposome layer on a filter (Flaten et al., 2006). PVPA was further improved by Engesland's work, which resulted in the production of a novel PVPA model which closely mimics the SC barrier of the skin (Engesland et al., 2013, 2015). This model was adopted by Palac et al. to study the effect of vesicle carrier on the skin penetration (Palac et al., 2014). In other efforts, Schurr and his team combined keratinocytes with degradable scaffolds to promote autologous healing (Schurr et al., 2012). Prior to this, Falanga et al. had attempted promoting autologous healing using allogeneic human fibroblasts (Falanga and Sabolinski, 1999).

The majority of skin substitute models are limited to an epidermal layer. These models can be significantly improved by integrating a dermal layer containing fibroblasts into the *in vitro* model. In the skin itself the interaction between fibroblasts and keratinocytes is fundamental to the wound healing process (Falanga et al., 2002). *In vitro* experiments demonstrated that the crosstalk between fibroblasts and keratinocytes promotes the keratinocytes growth by means of soluble growth factors (Groeber et al., 2011). Evidence from studies on skin substitutes have shown that fibroblasts also have a key role in the natural epidermal histogenesis and keratinocytes differentiation is greatly affected in the absence of fibroblasts (Boehnke et al., 2007; Groeber et al., 2011). Bell et al. were the first to describe such a complex model (Bell et al., 1981), which are referred to as full-thickness *in vitro* models. Many different techniques have since been used for the formation of such dermal layers (Parenteau et al., 1992; Sahuc et al., 1996; Stark et al., 2006). The extensive studies carried through within recent years have led to the commercial availability of many *in vitro* skin models (Boyce and Lalley, 2018) such as Apligraf™, StrataGraft™, DermaGraft™ (Frykberg et al., 2015), EpiCel™ (Sood et al., 2010), ReCell™ (Gravante et al., 2007), and TESTSKIN™ (Laska et al., 1992).

**Figure 8:**
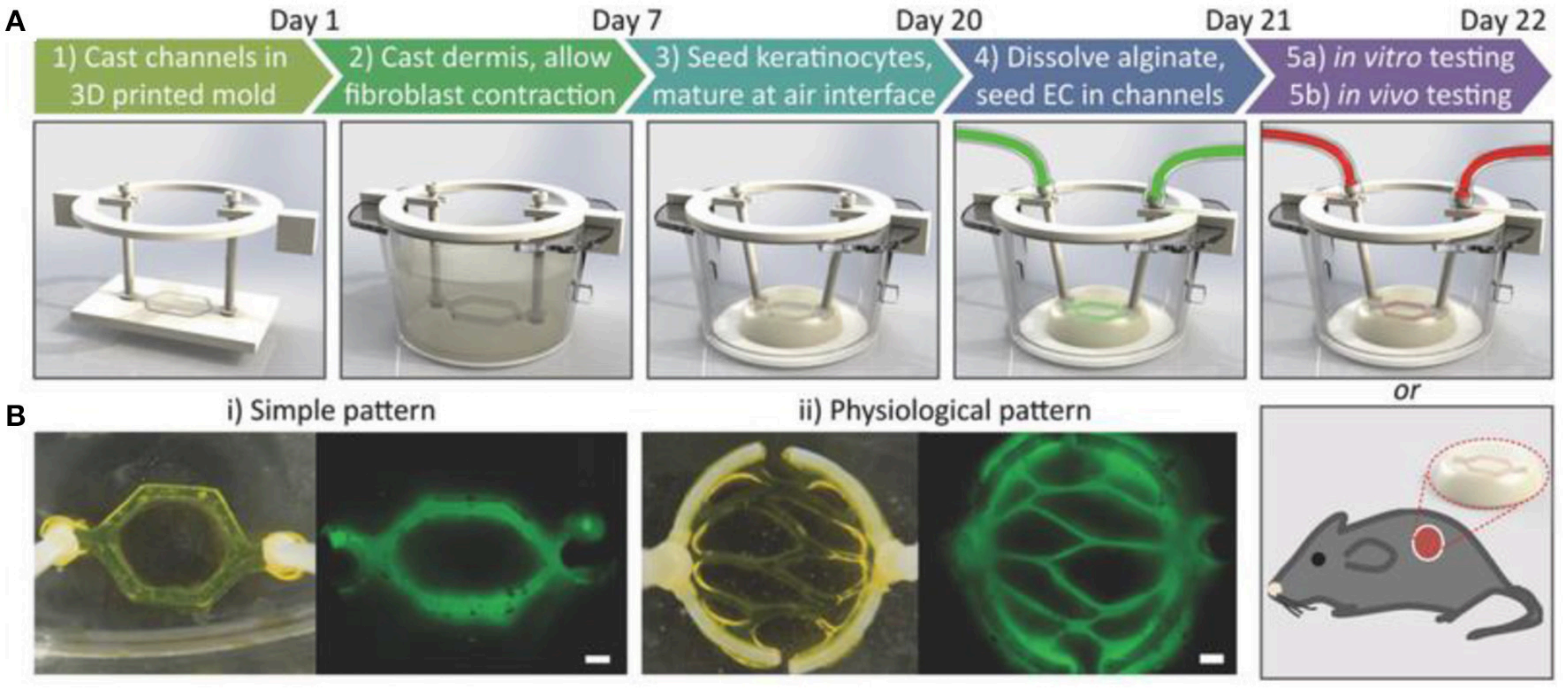
Development of vascularized human skin equivalents. **(A)** Schematic description of the protocol to develop human skin equivalents. **(B)** Two different vasculature patterns were generated using fluorescently tagged alginate. Scale bar: 600 μm. Reprinted with permission from Abaci et al. (2016). Copyright 2016, John Wiley & Sons.

Another emerging *in vitro* skin modeling approach is on-chip platforms which help to fabricate more physiologically relevant skin models for better understanding the underlying mechanism of skin diseases and discovery of new therapeutic agents. For example, a multi-organ-on-chip platform for skin and its appendages was fabricated using a multi-chamber microfluidics platform (Maschmeyer et al., 2015). The device was successfully tested for real-time immunohistological analysis and cell metabolic activity measurements. In another study, a simpler model containing bi-layer of keratinocytes-fibroblasts and endothelial cells-fibroblasts between three microfluidic channels was proposed to investigate penetration in skin (Wufuer et al., 2016). Furthermore, a simple full thickness skin-on-a-chip platform using a pumpless microfluidic device was developed to investigate pharmacokinetics of various substances (Abaci et al., 2015). To further mimic physiologically relevant skin model, a perfusable vascularized full-thickness model was also developed to mimic the dermis containing collagen seeded by induced pluripotent stem cells (iPSC) derived endothelial cells (Figure 8; Abaci et al., 2016). Overall, skin-on-chip models have shown great promise for substance testing, discovery and screening.

Despite fascinating advancements in the development of *in vitro* skin substitutes, there is still a long path laying ahead for full simulation of all functions and structures of the skin. Current major differences between skin substitutes and the skin itself include the absence of stable vascular and lymphatic networks, skin appendages (such as hair follicles, sweat glands and sebaceous) and hypopigmentation (Boyce and Lalley, 2018). The continuation of current developmental trends promises the correction of the remaining deficiencies in the near future, paving the path for complete replication of skin anatomy and physiology and further enhancement of skin disease and wound treatment.

### *In Vivo* models for skin substitute testing

Wound healing in human skin is an extremely complex process involving inflammation, re-epithelialization, granulation tissue formation, and dermal remodeling (Martínez-Santamaría et al., 2013). Finding accurate *in vivo* models for this process can be a challenge due to the high cost of *in vivo* work and the differences between animal models and human skin. Common *in vivo* skin tissue models for skin substitute testing include the guinea pig, mouse, rat, and pig. Table 1 summarizes these models and compares them based on their cost, thickness, hair follicle density, skin attachment, and the wound healing mechanism.

**Table 1:** Comparative properties and cost of *in vivo* wound healing models (Godin and Touitou, 2007; Gainza et al., 2015; Summerfield et al., 2015).

**Species**	**Thickness (mm)**	**Hair follicle density**	**Skin attachment**	**Wound healing mechanism**	**Cost**
Human	2.97	Low	Tight	Re-epithelialization	N/A
Guinea Pig	1-2	High	Loose	Contraction	Medium
Mouse	0.70	High	Loose	Contraction	Low
Rat	2.09	High	Loose	Contraction	Low
Pig	2.5	Low	Tight	Re-epithelialization	High

The guinea pig is an *in vivo* model in skin tissue engineering because, like human skin, guinea pig skin exhibits thick epidermal and dermal skin structure (Summerfield et al., 2015). Areas where the guinea pig is lacking in similarity to the human skin are skin-attachment, hair coat, and the healing mechanism. Guinea pigs have a contractile wound healing mechanism whereas humans heal wounds via re-epithelialization. Mice are another species that have been widely used for evaluating the performance and safety of skin substitutes, mainly due to their low cost. Similar to guinea pigs, mouse skin differs from human skin in that it is loosely attached, has a dense coat of hair, has a thin epidermis and dermis, and heals through contraction rather than re-epithelialization (Summerfield et al., 2015). Past research has gone into developing humanized mouse models, in which human keratinocytes and fibroblasts are grafted onto mouse skin to mimic human skin (Martínez-Santamaría et al., 2013). These types of humanized skin models have yet to be used for assessing the efficacy of other skin substitutes. In another study, the ability of a bioprinted skin substitute to differentiate was assessed in immunodeficient athymic mice with excised wounds (Cubo et al., 2016). It was found that the grafted area of skin closely resembled the structure and appearance of native human skin. This exemplifies the usefulness of mice as an *in vivo* model in developing a fully differentiated form of human skin.

Another commonly used *in vivo* model for skin substitute testing are rats. Similarly to mice, they have loosely attached skin, dense hair, and they heal through contraction (Summerfield et al., 2015). Rats offer an affordable *in vivo* model that are widely used for modeling wounds of various types. For example, Sprague-Dawley rats were used to model third degree burn wounds in developing a skin substitute made of collagen seeded with genetically modified immortal keratinocyte cells (Hu et al., 2012). However, there are large differences in the speed at which rats heal when compared to humans and that further investigation is required to verify the potential efficacy for the treatment of human wounds.

Pigs have the most similarities to human skin in that it is firmly attached, has sparse hair follicle distribution, has similar epidermal and dermal thickness, and heals through the process of re-epithelialization (Summerfield et al., 2015). On top of these structural and wound healing similarities, porcine skin also has a similar blood supply and immunological function. The limiting factor for the use of pigs as an *in vivo* model for most researchers is the high cost due to their difficulty in handling, longer gestation time, and large space requirements (Gainza et al., 2015). However, due to their large size, a single pig can act as a model for multiple wound sites. In a study by Shevchenko et al. pigs were chosen for their similarities with human skin and each pig modeled 6 separate wound sites to assess the efficacy of a gelatin scaffold as a dermal replacement (Shevchenko et al., 2014). However, their animal study did not go as planned and the pseudo-epidermal silicone layer on their models peeled off, exposing their gelatin dermal scaffolds. They did not repeat the experiment and speculated, based on their *in vitro* results that their treatment has the potential to accelerate wound healing in human skin. This exemplifies the importance of being completely prepared before conducting expensive *in vivo* testing.

## Roadmap for biomaterial selection, risk assessment and testing

Any medical device and therapeutic strategy should first pass some regulatory obligations to reach the market. Therefore, devices are categorized as Class I, II, or III based on the level of regulation required to guarantee their safety and efficacy (Morrison et al., 2015). Low-risk Class I devices only undergo general and simple regulatory controls, while high-risk Class III devices (e.g., most implants), are subjected to the most rigorous regulations. The latter are usually permitted an initial Investigational Device Exemption (IDE) to be employed in a FDA-regulated clinical trial to gather required safety and efficacy data prior to market application. These devices can obtain premarket approval (PMA) pathway for commercialization (Morrison et al., 2015).

Skin substitutes are regulated by the regulating agency in different regions of the world including the US Food and Drug Administration (FDA), Health Canada and regional and centralized regulatory bodies in the European Union (EU) (Van Norman, 2016). Full thickness skin grafts combine scaffolds, multiple cell types and sources. As each of these elements must be regulated, a lengthy and complicated regulatory process has been enforced for the commercialization process. For example, different regulatory centers are involved to evaluate, review and register a new skin graft in the USA including: The Center for Biologics Evaluation and Research (CBER), The Center for Devices and Radiologic Health (CDRH), and The Center for Drug Evaluation and Research (CDER).

Biomaterials that are used in fabrication of the skin grafts must be evaluated according to the FDA Quality System Regulation (QSR)/Medical Device Good Manufacturing Practices (GMP) for industry manufactured devices (Lincoln, 2010). Furthermore, the cells that undergo culture, expansion, and/or differentiation, or combined with biomaterial scaffold must meet the CBER premarket approval requirement (PMA). On the other hand, if a graft is fabricated by the emerging manufacturing technologies (e.g., 3D printing, electrospinning, microfluidics, etc.), a number of quality measures have to be taken into account to ensure repeatability of the process and reliability of the grafts. This data could facilitate the review process by the regulating bodies and expedite the PMA pathway.

The FDA has been proactively involved in communicating with different bodies in research and development settings in industries and academics to define a clear roadmap for commercialization of a medical device to the market. For example, tissue-engineered medical products (TEMPs) are being reviewed by a specific collaborative commission with the FDA through the American Society for Testing and Materials (ASTM). The mandate of this commission is to define new materials and provision of standard methods for calibration and testing of these materials including tissue engineered skin grafts according to the “Standard Guide for Classification of Therapeutic Skin Substitutes.”

In addition, the Office of Combination Products review the new combination therapies and endorse their safety and efficacy in collaboration with different centers (e.g., Centers for Human Therapeutics) and regulating offices. This office assigns the premarket review and evaluation of a combination product based on determination of its primary mode of action (PMOA). For example, if the main PMOA of a combination product attributes to the biological product, the related agency responsible for the biological product will have primary role in regulating the combination product. However, if the PMOA of a combination product is diverse, the regulating agencies have to take the difficult and extensive decision (i.e., trade-off between rapid access to novel products for the patients in urgent need and appropriate promises on safety and efficacy) about which agency is responsible for reviewing and regulating this specific therapy.

**Figure 9:**
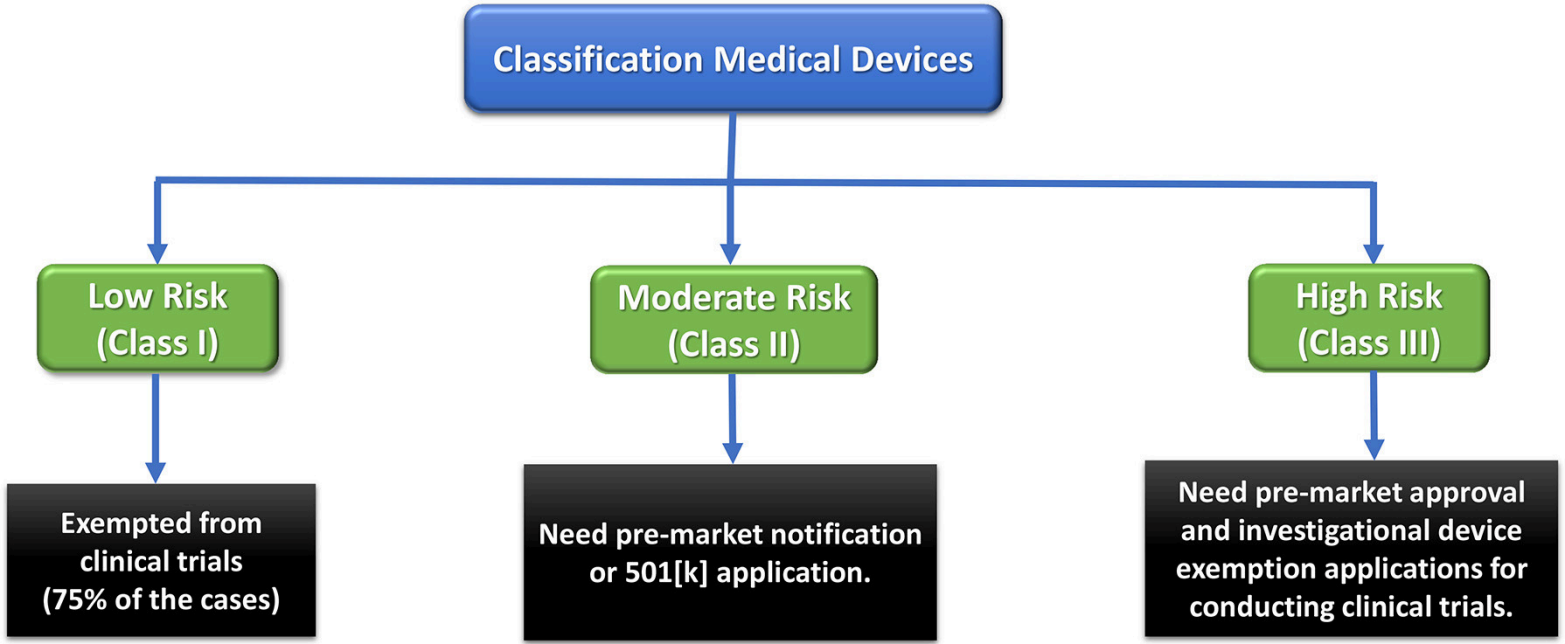
Schematic of FDA medical device approval roadmap.

Although extensive efforts have been made to clarify the medical devices regulatory pathway to the market, there has been an unmet need to expedite and facilitate this process in order to provide novel therapeutic strategies for patients with life-threatening diseases. To this end, the FDA has mandated some coherent and flexible designations to address the lengthy regulating process for the innovative medical devices including Fast Track development, Breakthrough Therapy designation, Accelerated Approval and Priority Review designation for drugs. In addition to the aforementioned, the most recent Regenerative Medicine Advanced Therapy (RMAT) designation based on the twenty-first Century Cures Act (Cures Act) has the mission to address the lengthy regulating process for innovative medical devices. Figure 9 shows FDA medical device approval roadmap.

## Conclusion and future perspective

Tissue engineering of skin is a well-established but growing field in regenerative medicine. There have been tremendous efforts to employ emerging micro- and nano-fabrication strategies, biomaterials synthesis, functionalization techniques and patient specific cells' utilization to fabricate remarkable potential functional skin substitutes that could tackle the challenges facing currently available skin grafts. For example, there have been many reports on resembling of the ECM with combining nontoxic immunomodulatory biomaterials, growth factors, proteins and biomolecules along with the advanced processing strategies. Although many newly synthesized biomaterials have been investigated as the scaffolds in wound dressings, only natural biopolymers such as collagen, gelatin, and chitosan have been extensively used for the commercial skin grafts (Sheikholeslam et al., 2017). However, these materials suffer from low mechanical stiffness and fast degradation which limits their applications in clinics. On the other hand, synthetic biomaterials such as poly vinylpyrrolidone (PVP), PCL, poly ethylene glycol (PEG), poly lactic acid (PLA) possess promising mechanical properties including elasticity and contractibility, similar to those of native skin with less biocompatibility and *in vitro* and *in vivo* functionality and performance. Therefore, researchers have attempted to address these issues surrounding tuning mechanical and structural properties of wound dressings by synthesizing novel elastomeric biodegradable biomaterial and/or optimizing the properties of the existing biomaterials.

However, there are yet unresolved complications such as wound contraction, impaired vascularization, scaring, and high cost associated with these products that need to be carefully addressed (Ho et al., 2017). Vascularization is vital for the success of artificial skin grafts which leads to increased life span and better integration with host skin. *In vivo* vasculogenesis of the grafts can be promoted by incorporating cells such as endothelial cells and mesenchymal and adipose-derived stem cells to the scaffolding materials (Jackson et al., 2012; Marino et al., 2014), using angiogenic biomolecules (Briquez et al., 2015) and tuning the structural properties of the scaffolds (Bonvallet et al., 2015). On the other hand, application of the cell-based skin substitutes has been limited in clinics due to the time consuming and labor-intensive process and short shelf-life of the products. Therefore, *in situ* regeneration could be a promising alternative in the near future. The advancement of innovative fabrication techniques such as in situ electrospinning and 3D printing and microfluidics along with the emergence of the new functional biomaterials could provide the on-demand fabrication of skin substitutes that are tailored to a patient's wounds. It is possible to recruit stem cells and progenitor cells from the wound site by using bioactive materials with suitable morphology *in situ*, to encourage migration/infiltration of the residing cells and differentiation of stem cells into favorite cell types and finally regenerate newly-formed functional skin.

Translation of such artificial skins to the clinics, manufactured with the novel technologies stated above, needs predictive test methods and appropriate standards and regulations to ensure the reproducibility and functional reliability of the grafts.

In general, optimal functional skin substitutes need to possess the improved adhesion of cultured keratinocytes to the wound bed, improved neovascularization and enhanced resistance to the wound contraction and fibrosis. Although these criteria have almost been addressed by the exhaustive efforts during the past decades, other complicated challenges such as reconstruction of skin appendages, thermoregulation, touch, excretion and the esthetic function remain to be solved.

## Author contributions

MA is the corresponding author of this work. He developed the outline with HS edited the paper. HS prepared the outline, led the project and wrote the paper. BG and MH wrote the manuscripts.

### Conflict of interest statement

The authors declare that the research was conducted in the absence of any commercial or financial relationships that could be construed as a potential conflict of interest. The reviewer JB and handling Editor declared their shared affiliation.
